# Multipopulation Whale Optimization-Based Feature Selection Algorithm and Its Application in Human Fall Detection Using Inertial Measurement Unit Sensors

**DOI:** 10.3390/s24247879

**Published:** 2024-12-10

**Authors:** Haolin Cao, Bingshuo Yan, Lin Dong, Xianfeng Yuan

**Affiliations:** School of Mechanical Electrical and Information Engineering, Shandong University, Weihai 264209, China; haolincao@mail.sdu.edu.cn (H.C.); yanbingshuo@mail.sdu.edu.cn (B.Y.); donglin@sdu.edu.cn (L.D.)

**Keywords:** feature selection, whale optimization algorithm, multipopulation, human fall detection

## Abstract

Feature selection (FS) is a key process in many pattern-recognition tasks, which reduces dimensionality by eliminating redundant or irrelevant features. However, for complex high-dimensional issues, traditional FS methods cannot find the ideal feature combination. To overcome this disadvantage, this paper presents a multispiral whale optimization algorithm (MSWOA) for feature selection. First, an Adaptive Multipopulation merging Strategy (AMS) is presented, which uses exponential variation and individual location information to divide the population, thus avoiding the premature aggregation of subpopulations and increasing candidate feature subsets. Second, a Double Spiral updating Strategy (DSS) is devised to break out of search stagnations by discovering new individual positions continuously. Last, to facilitate the convergence speed, a Baleen neighborhood Exploitation Strategy (BES) which mimics the behavior of whale tentacles is proposed. The presented algorithm is thoroughly compared with six state-of-the-art meta-heuristic methods and six promising WOA-based algorithms on 20 UCI datasets. Experimental results indicate that the proposed method is superior to other well-known competitors in most cases. In addition, the proposed method is utilized to perform feature selection in human fall-detection tasks, and extensive real experimental results further illustrate the superior ability of the proposed method in addressing practical problems.

## 1. Introduction

With the continuous development of science and technology, the volume of data is increasing exponentially, which creates “the curse of dimensionality” [1]. In the domain of feature selection, researchers have introduced numerous approaches to address the diversity and complexity of data characteristics. However, many features of the vast databases are nonessential or even redundant. To cope with this issue, researchers have turned to feature selection (FS) as a common tool. By removing irrelevant and redundant features, FS obtains an optimal subset with very few features and high classification accuracies to achieve dimensionality reduction [2]. There are applications of FS techniques in many fields, including text classification [3], bioinformatics [4], spectral-spatial classification [5], and others.

FS has four main operations, generating feature subsets, evaluating feature subsets, stopping criterion, and evaluation [6]. The search methods and evaluation criterion are the most essential parts of the FS algorithms. There are three main search strategies, a complete search strategy, sequence search strategy, and heuristic search strategy. A complete search necessitates traversing all the potential feature subset combinations, such as an exhaustive search [7]. While this strategy assures that the optimal subsets can be obtained, it comes with a considerable computational cost for large datasets. Based on model assessment feedback, the sequence search continuously adds or removes features from the candidate feature subsets to form the best feature subset. Common approaches are sequential backward selection (SBS) [8], sequential forward selection (SFS) [9], sequential floating backward selection (SFBS) [10], and sequential floating forward selection (SFFS) [11]. Lakshmi and Mohanaiah proposed a Multi-Support Vector Neural Network (MultiSVNN) method for FER based on a hybrid Whale Optimization Algorithm-Teaching Learning Based Optimization (WOA-TLBO) [12]. The sequence search algorithms reduce computing costs effectively, but it is easy to fall into the local optimum. A heuristic search gradually employs metaheuristics to approximate the global best subset. The metaheuristics can thoroughly consider the feature interactions during exploration and find the position closer to the target. Hence, a heuristic search is better suited than the first two methods for dealing with complicated and high-dimensional real-world problems. These heuristic algorithms include adaptively balanced grey wolf optimization [13], bare-bones particle swarm optimization [14], and a cultural algorithm and differential evolution [15], etc.

The Whale Optimization Algorithm (WOA) is one of the typical heuristic algorithms that is designed based on the feeding process of humpback whales [16]. The WOA is known for its efficient local exploitation ability and only needs to tune two main internal parameters. With these benefits, WOA has been implemented in a range of areas, including facial emotion recognition [17], medical diagnosis problems [18], and feature selection [19]. To achieve superior feature combinations, researchers frequently improve WOA from many perspectives. To tackle the challenge of feature selection in imbalanced classification, Lin Sun and colleagues proposed a two-stage feature subset selection scheme that integrates the Fuzzy Multi-Granularity Rough Set (FMRS) and a Binary Whale Optimization Algorithm (BWOA) [20], for instance, maintaining the balance of exploration and exploitation [21], getting rid of the local optimum [22,23], improving the global search ability [24,25,26], speeding up convergence [27], and promoting population diversity [28].

This study focuses on three aspects of the challenges in WOA-based feature selection. First, the global best individual of a canonical WOA is the primary directing factor in the search process. Individuals may likely cluster together early in this instance, resulting in limited global exploration capabilities and a premature convergence. Second, as the distance between individuals gradually decreases, the original search approach cannot guarantee that individuals continue to explore alternative locations, which could cause the search to stall. Finally, the population explores everywhere, and individual quality is poor in the initial stage, leading to low search efficiency.

In the context of fall detection, our proposed algorithm demonstrates the effectiveness of the feature selection (FS) method while primarily contributing to advancing FS research. Koo et al. introduced an artificial neural network (ANN) approach integrating ranking algorithms for post-fall detection, which enhances classification performance and balances accuracy with computational efficiency [29]. Their innovative use of ranking algorithms significantly improves dimensionality reduction, offering valuable insights for FS optimization. Similarly, Le et al. compared machine learning classifiers in fall detection, showing that FS techniques improve model performance, reduce computational complexity, and refine feature sets [30]. This study highlighted the synergistic effects of classifiers and FS methods, providing critical guidance for addressing fall-detection challenges. Moreover, widely adopted datasets, such as those in [31] and the influential dataset by Jang et al. [32], have significantly supported research efforts. These works underscore the importance of effective FS and robust datasets in advancing fall-detection applications and FS research.

Therefore, a multispiral whale optimization algorithm (MSWOA) with three improvements is presented to enhance the global search capability and jump out of the local optima trap. The following are the key theoretical contributions of this paper:

- A new Adaptive Multipopulation merging strategy (AMS) separates a population into several subpopulations based on a particular exponential function, which can generate more candidate solutions and expand the exploration space. Each subpopulation selects individuals according to a fitness-distance criterion, which enhances global exploration capability and prevents premature convergence.
- A Double Spiral updating Strategy (DSS) based on the golden spiral is designed to update the position of individuals along a specific spiral path. The DSS and the bubble-net attacking method use different spiral properties to achieve inward exploitation and outward exploration, which can address the issue of stalling to the local optimum solutions.
- A Baleen neighborhood Exploitation Strategy (BES) combines quadratic interpolation (QI) with baleen-assisted predation behavior to enhance the candidate solutions and accelerate convergence. This strategy explores more prospective areas by focusing on the area near the best location, which provides a good direction for the population in the early optimization process.
- The performance of the MSWOA-based feature selection algorithm is tested on 20 UCI classification datasets and a realistic classification optimization problem.
- The MSWOA is tested against six variants of the WOA and six other advanced swarm intelligence optimization approaches. Extensive feature selection experiments indicate that MSWOA obtains a more promising overall performance compared with the comparison algorithms on most datasets.

The remaining organization of this paper is as follows: Section 2 provides the various works related to feature selection. Section 3 describes an introduction to the original WOA. Section 4 details the improvement points of the MSWOA approach. Section 5 compares MSWOA with other algorithms and analyzes the reported results from the experiments on various benchmark datasets and real-world problems. Section 6 summarizes this paper and suggests the subsequent research options.

## 2. Related Work

The feature subset chosen using feature selection approaches contains valuable information, which reduces the training time and facilitates the classification accuracy. Thus, FS can cope with the challenges of high-dimensional data with large quantities of information and complex feature interaction. FS approaches are broadly categorized into filter-based, wrapper-based, and embedding-based methods [33].

Filter-based methods sort features according to different criteria and then select features that meet the threshold and generate a feature subset. This method is independent of the classification algorithm, which gives it the advantage of being computationally efficient. There are several commonly used filtering methods. Eiras-Franco et al. [34] described a new version of ReliefF that simplifies the most expensive phase by approximating the nearest neighbor graph with locality-sensitive hashing (LSH). Song et al. [35] presented a new feature ranking approach on the basis of Fisher discriminant analysis (FDA) and an F-score, which obtained a suitable subset by maximizing the proportion of the average between-class distance to the relative within-class scatter. Other improved approaches in this category include minimal redundancy maximal new classification information (MR-MNCI) [36] and information gain (IG) [37]. However, the selected feature subset using filters has low prediction accuracies due to the lack of a specific learning algorithm guiding the feature selection phase [38].

Wrapper-based methods introduce the learning algorithm to evaluate the quality of feature subsets and consider the complex interactions between the features. A learning algorithm is used in each iteration to assess whether the candidate feature subset is superior to the current best subset [39]. As learning algorithms for evaluating features, the decision tree (DT), K-nearest neighbor (KNN), naïve Bayes (NB) and support vector machine (SVM) have been frequently utilized. In this case, wrappers have better classification accuracy than filters. Recursive feature elimination (RFE) is a traditional wrapper FS method that employs a machine learning model for training and constantly removes features based on the weight value. Han et al. [40] presented a dynamic recursive feature elimination (dRFE) framework. Another typical wrapper feature selection method is the Las Vegas wrapper (LVW) [41]. However, the adoption of learning algorithms incurs a considerable computational cost if the search space is vast [42]. The conventional optimization techniques that search for the optimal feature subset becomes extremely difficult. Therefore, with their powerful global search ability, metaheuristic approaches have been successfully employed in feature selection, these include the ant colony optimization (ACO) and the grasshopper optimization algorithm (GOA).

Embedded-based approaches combine the model construction of the learning algorithm with the feature selection process. The regularization method is one of the most common embedded methods. Based on multimodal neuroimaging, Zhang et al. [43] introduced a new multiclass categorization framework for Alzheimer’s disease (AD) with the introduction of feature selection and fusion, which used a l2,1-norm regularization term and a lp-norm (1 < *p* < ∞) regularization term. Other algorithms also include the particle swarm optimization-based multiobjective memetic algorithm (PSOMMFS) [44], classification and regression trees (CARTs) [45], decision tree ID3 algorithm [46], etc. Although embedded methods have good classification performance and efficiency, their approach structure is more complicated and ignores feature interactions. Therefore, this paper utilizes a wrapper method based on a metaheuristic algorithm to address the feature-selection problem.

Among the existing approaches, metaheuristics are employed in feature selection in various forms, with hybrid algorithms being one of the most used. Amini and Hu [47] presented a novel hybrid two-level FS method, which combines a GA and EN to construct an appropriate subset of predictors to improve computational efficiencies and prediction performances. Ibrahim et al. [48] integrated the SSA with PSO to boost exploitation abilities and diversity, helping to swiftly obtain the optimal feature subset. This paper presented a novel algorithm for finding high-quality solutions for feature-selection tasks by incorporating the SSA into the Harris hawks optimization (HHO) algorithm [49]. Additionally, academics frequently introduce different strategies in metaheuristics to improve classification performance. Li et al. [50] suggested an enhanced PSO algorithm for feature selection. The personal best position was updated with genetic operators in this technique to avoid the local optimum. Chen et al. [51] presented a novel PSO-based feature selection approach that uses information about the swarm to produce more potential solutions. An improved discretization-based PSO for FS (IDPSO-FS) in [52] was developed to identify each selected feature index accurately. The authors of [53] suggested an improved version of the salp swarm algorithm (ISSA) to solve feature selection problems and to select the optimal subset of the features in wrapper mode. In [54], an improved binary global harmony search algorithm (IBGHS) with a modified improvisation step was presented. Furthermore, several recent articles have combined filter approaches and metaheuristic-based wrapper approaches. Ghosh et al. [55] developed a wrapper-filter combination on the basis of ACO, in which a filter approach is used to evaluate a subset to reduce the computing complexity. In the same year, Moslehi and Haeri [56] presented a hybrid filter-wrapper method that integrates evolutionary-based GA and PSO; an artificial neural network was included into the fitness function.

The WOA is an evolutionary algorithm with simple principles and an easy implementation. Moreover, the WOA exhibits superior performance in solving FS problems. Tubishat et al. [57] incorporated IG filter-feature-reduction techniques with the WOA to capitalize on its advantages and compensate for its shortcomings. The IWOA used the EOBL technique to improve the diversity of the initial solutions and DE evolutionary operators to improve the solutions after each iteration. The IWOA outperformed the other approaches (i.e., WOA, DE, PSO, GA, ALO) on four datasets. To achieve automatic lung tumor detection, a hybrid bioinspired algorithm (WOA_APSO) was developed that combined the benefits of the WOA and APSO to select the optimized feature subset [58]. The experimental results revealed that it outperformed the other advanced methods and achieved 97.18%, 97%, and 98.66% accuracy, sensitivity, and specificity, respectively. In [59], a wrapper-based FS method that hybridized GWO and WOA was developed, aggregating the advantages of their exploration and exploitation. The results showed that HSGW outperforms several well-known feature selection methods (i.e., BGOA, BGSA, GA, and PSO). Mafarja et al. [60] presented a new WOA-based FS by introducing both V-shaped and S-shaped transfer functions, which were adopted in IoT attack detections. This paper integrated five natural selection operators into WOA to enhance the search efficacy in dealing with FS tasks [61]. The proposed wrapper approach was used to determine the most valuable features from the software fault prediction (SFP) datasets. To find the best subset, two binary variants of the WOA were presented [62], which used the sigmoid transfer function and the sigmoid transfer function, respectively. There is also a substantial body of research describing the different WOA variants used in feature selection [63,64,65].

Based on the no-free lunch (NFL) theorem [66], no single strategy can solve all the WOA issues. Thus, there is still some room for further improvement. Focusing on the problem of insufficient global search capability and frequent search stagnation, this paper proposes an innovative wrapper approach called the multispiral whale optimization algorithm (MSWOA). We divide the population into many subpopulations using the novel multipopulation technique, which may enlarge the search space and yield more candidate subsets. In addition, a new stochastic exploration strategy is provided to assist individuals in breaking free from local optima. Furthermore, to maximize the quality of the optimal individual, we apply an exploitation method based on humpback whale behaviors. The MSWOA is tested on 20 UCI datasets and in two group contrast trials. The results show that MSWOA can obtain feature subsets with higher classification accuracies and fewer features on most datasets. Section 4 will go through the MSWOA in further detail.

## 3. The Whale Optimization Algorithm (WOA)

The WOA is inspired by the distinctive hunting activity of humpback whales. Humpback whales engage in an interesting hunting method called bubble-net feeding, which involves blowing bubbles around the prey in a spiral or ‘9’-shaped route and pushing it toward the center of the net. In the WOA, the target prey symbolizes the current best solution, and humpback whales are the search agents. Based on these characteristics, the mathematical model of WOA is divided into three components: encircling prey, the bubble-net attacking method, and searching for the prey.

### 3.1. Encircling Prey

When humpback whales swim around their prey, they shrink the circle while swimming in a spiral pattern. After targeting the prey, the search agents keep coming closer to the best solution. The mathematical model of how the present search agent adjusts its location based on the best record position is shown in Equations (1) and (2).

(1) $$D=\left|C\cdot X^{*}(t)-X(t)\right|$$

(2) $$X(t+1)=X^{*}(t)-A\cdot D$$

where *t* indicates the current iteration, X* indicates the position of the global optimal individual, and *X* denotes the position of the current individual. | | indicates the absolute value, and · indicates an element-by-element multiplication. The values of vectors *A* and *C* control the movement of the current individual around the best solution, and their updating equations are as follows:

(3) A=2a⋅r−a

(4) C=2⋅r

where *r* is a randomly chosen vector in [0, 1]. The range of a is reduced from 2 to 0 by Equation (5).

(5) $$a=2-t\frac{2}{MaxIter}$$

where *t* denotes the current number of iterations and *MaxIter* denotes the number of maximum iterations.

### 3.2. Bubble-Net Attacking Method

The spiral updating position simulates the unique mechanism of bubble-net foraging to move toward the current optimal solution. The spiral equation is as follows:

(6) $$X(t+1)=D^{\prime }\cdot e^{bl}\cos(2\pi l)+X^{*}(t)$$

(7) $$D^{\prime }=\left|X^{*}(t)-X(t)\right|$$

where *D′* is the distance of the *i*th search agent from the optimal individual. *b* is a constant, and *l* is a randomly chosen number in [−1, 1].

Above all, the model assumes a parameter *p* to choose between either encircling prey or using bubble-net attacking to update the position of the whales as in Equation (8).

(8) $$X(t+1)=\left\{\begin{array}{ll} X^{*}(t)-A\cdot D & if\;p<0.5\\ D^{\prime }\cdot e^{bl}\cdot \cos(2\pi l)+X^{*}(t) & if\;p\geq 0.5 \end{array}\right.$$

where *p* is a random number in [0, 1].

### 3.3. Search for Prey

The exploration phase imitates the behavior of humpback whales when searching randomly. The mathematical formulations of this mechanism are shown in Equations (9) and (10).

(9) $$D=\left|C\cdot X_{rand}-X(t)\right|$$

(10) $$X(t+1)=X_{rand}-A\cdot D$$

where *X_rand_* is a randomly selected individual from the current swarm. The absolute parameter value |*A*| determines whether the algorithm emphasizes exploitation or exploration. When |*A*| ≥ 1, Equation (10) is selected to force a solution to explore the potential space. When |*A*| < 1, Equation (2) is selected.

The pseudocode of the WOA algorithm (Algorithm 1) is as follows:
**Algorithm 1.** WOA1 Initializing the population *X_i_* (*i* = 1, 2…, *n*)2 Calculate the individual fitness values in turn3 X* = the best individual4 **while** (*t* < *MaxIter*)5  **for** each individual6    Update *a*, *l*, *p*, *A*, and *C*7    If (*p* < 0.5)8       If (|*A*| < 1)9         Calculate the new position of the current individual according to Equation (2)10      else if (|*A*| > 1)11        Randomly select a individual (*X_rand_*) 12        Calculate the new position of the current individual according to Equation (10)13      end 14    else if (*p* > 0.5)15      Calculate the new position of the current individual according to Equation (6)16    end 17  **end**18  If a search agent exceeds the search range, correct it19  Calculate the individual fitness values in turn20  Update X* if there are better results21  *t* = *t* + 122 **end**23 return X^*^

## 4. Proposed MSWOA

Although the WOA achieves good performance in finding reasonable solutions for feature-selection tasks, it still has some drawbacks, including limited global exploration capability and a proclivity to be trapped in the local optimum. To address the drawbacks of WOA, this paper proposes a multispiral whale optimization algorithm, namely, the MSWOA algorithm, which mainly contains three major modifications, an Adaptive Multi-population merging Strategy (AMS), a Double Spiral updating Strategy (DSS), and a Baleen neighborhood Exploitation Strategy (BES). The contents of the three improvement strategies are described below.

### 4.1. Adaptive Multipopulation Merging Strategy (AMS)

The leader of the population in the original WOA algorithm is single, which might lead to poor global exploration capability and premature convergence. According to Equation (8), the population individuals update their positions primarily according to X* with the best fitness. Therefore, the search range is relatively limited in comparison to the entire space. For this problem, a multipopulation mechanism is a workable approach, in which the population is divided into several subpopulations to enhance the possibility of exploring more space [67]. Each swarm follows its own leader to search for different areas in the search space, which effectively improves the global exploration abilities. Due to the benefits listed above, we introduce the multipopulation concept into the WOA and redesign a more reasonable subpopulation-allocation process with new strategies.

The first procedure is to count the number of subpopulations. In the initial stages of evolution, subpopulations with smaller sizes are advantageous to the diversity of the swarm [68]. In contrast, larger subpopulations are more favorable for producing high-quality solutions in the later stages of evolution. Hence, the number of subpopulations needs to be smoothly merged from K to one. The calculation formula uses exponential characteristics, as shown in Equation (11).

(11) $$K=floor(\frac{N}{2}\cdot \exp(-\ln(\frac{N}{2})\cdot \frac{t}{MaxIter})+\gamma )$$

where *K* is the number of subpopulations, *floor* represents a function that rounds toward minus infinity, *N* is the population size, *t* is the current number of iterations, and *MaxIter* is the number of maximum iterations. *γ* is a parameter in [0, 1] that is chosen based on the size of the population. Therefore, each subpopulation size is determined as *M = floor*(*N/K*) according to this selection scheme.

**Figure 1:**
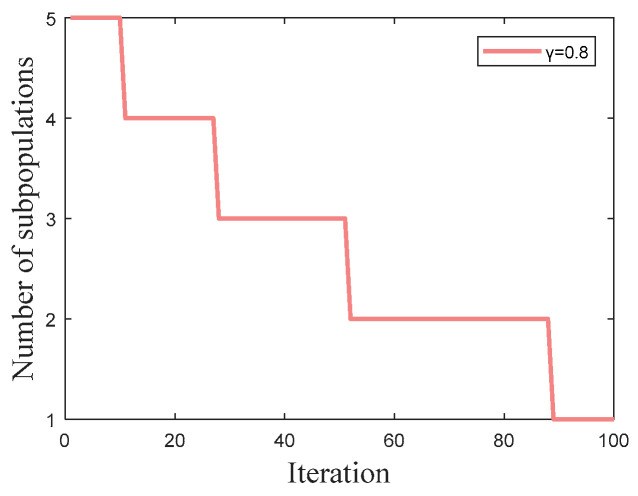
Variation curve of the number of subpopulations.

For example, when *N* is 10 and *MaxIter* is 100, it is reasonable to set *γ* to 0.8, and the change curve of the number of subpopulations is illustrated in Figure 1. The convergence rate of the number of subpopulations is faster in the early iterations and then gradually become slower, ensuring enough space for exploration in the early stages and finally merging together for deeper exploitation. In the initial phase, the number of subpopulations is five, with each subpopulation containing two individuals. Each subpopulation explores different areas under the guidance of its own optimal individual. Soon, the number of subpopulations aggregates to four, after which they progressively merge into three or two populations at a modest rate. Finally, all individuals aggregate into a single population for a deeper exploitation. This kind of regulation effectively balances exploration and exploitation while providing continuous information sharing.

The second procedure is the selection of the subpopulation individuals. Generally, individuals with different fitness levels have different exploration and exploitation abilities [69]. In addition, taking into account the distance between individuals contributes to enhancing diversity. Therefore, a new selection mechanism is defined in Equation (12), which combines the fitness value and the distance between individuals. According to the comprehensive calculation results from high to low, all the individuals are categorized into different subpopulations.

(12) $$FD_{i}=(1-fit_{i})+dis_{i},\;i=1\;,\;2\ldots ,\;N$$

where *fit* is the normalized fitness, and *dis* is the normalized value of the distance between the search agent and the global optimal search agent.

In addition, the AMS strategy also introduces a randomized regrouping schedule as another selection mechanism. Every *R* iterations, individuals are randomly selected for each subpopulation. This mechanism exchanges information among the swarms to stimulate more vitality in the individuals and achieves a wider diversity. The flow of the proposed AMS is shown in Algorithm 2.
**Algorithm 2.** AMS **Input:**
*N*, *R*, *t*1 Calculate the number of subpopulations *K* by Equation (11) and the size of subpopulations *M* = floor(*N*/*K*)2 Calculate the individual fitness values in turn3 X* = the best individual 4 Update FD by the Equation (12) and reorder the population5 If mod (*t*, *R*) = 06   Randomly select individuals to form subpopulations7 end 8 Each subpopulation selects *M* particles in order  **Output:** subpopulations *X_m_(1* ≤ *m* ≤ *K)*, *M*, *K*

### 4.2. Double Spiral Searching Strategy (DSS)

In the exploration process of the original WOA, a search agent mainly relies on randomly selected individuals to update its location. As shown in Equation (10), when the distance D is small, it is difficult to obtain a competitive new solution. The search is very likely to stop, especially in the later stage of the iteration process. Therefore, the search strategy is further improved by using the golden spiral in this scenario. A golden spiral, such as WOA’s bubble-net attacking method, can spiral inward indefinitely and has a strong exploitation ability. In addition, it can also spiral continuously outward. According to this characteristic, the search agent starts from its current place and then spirals randomly to any location nearby or far away. The position update formulas are defined as follows:

(13) $$X_{m}(t+1)=r\cdot e^{\theta }\cdot \cos(2\pi \theta )+X_{m}(t)$$

(14) $$r=\left|W\cdot X_{rand}-X_{m}(t)\right|$$

where *X_m_* indicates the current position in the *m*th subswarm, *X_rand_* indicates a random agent of another subswarm, *θ* controls the radian of the helix, and *W* is a random vector in [0, 1].

**Figure 2:**
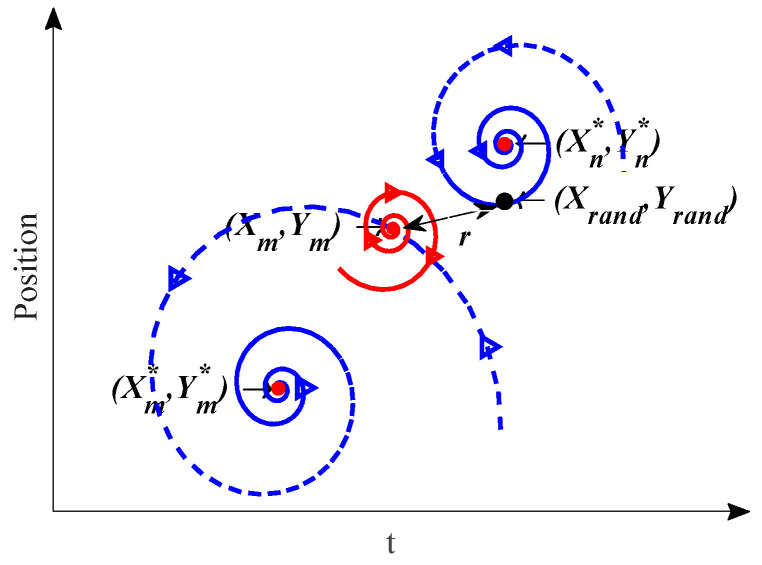
Particle spiral trajectory curve.

After implementing the AMS and DSS techniques, the search path has many interacting helices. Part of the population during optimization is depicted in Figure 2. The current agent is located at (*X_m_*, *Y_m_*), which is one of the *m*th subpopulation. Randomly select an agent (*X_rand_*, *Y_rand_*) in the population that belongs to the *n*th sub-population. *r* represents the helix radius of the particle update. According to Equation (13), the agent can explore any place in the population spiral gap, which increases the randomness and exploration ability considerably.

### 4.3. Baleen Neighborhood Exploitation Strategy (BES)

Humpback whales have dozens of baleens to assist them in discerning if there is prey nearby, which could be thought of as a local search. Inspired by this idea, a novel Baleen neighborhood Exploitation Strategy (BES) is presented, which imitates baleen behavior with a quadratic interpolation (QI) to speed up convergence. QI is a local search operator that explores the extremum using quadratic approximation [69]. The new method can help the optimal search agent purposefully search for better positions inside the neighborhood. It is beneficial in the early stages of iteration to overcome the poor fitness of individuals. The calculation formula necessitates the determination of three individuals. In this paper, select the optimal individual X_m_* (*g*_1_, *g*_2_…, *g_D_*) and an individual *X_m_* (*m*_1_, *m*_2_…, *m_D_*) in the mth subswarm, and the last one *X_r_* (*r*_1_, *r*_2_…, *r_D_*) is chosen at random from the population.

(15) $$\begin{array}{l} q_{j}=\frac{\left(m_{j}^{2}-r_{j}^{2})\cdot F(X_{m}^{*})+(r_{j}^{2}-g_{j}^{2})\cdot F(X_{m})+(g_{j}^{2}-m_{j}^{2})\cdot F(X_{r}\right)}{2\cdot ((m_{j}-r_{j})\cdot F(X_{m}^{*})+(r_{j}-g_{j})\cdot F(X_{m})+(g_{j}-m_{j})\cdot F(X_{r}))}\\ \;j=1,\;2\ldots ,\;D \end{array}$$

where *q_j_* is the *j*th dimension of the new solution *X_q_* (*q*_1_, *q*_2_…, *q_D_*). *F*(X_m_*), *F*(*X_m_*), and *F*(*X_r_*) are the fitnesses of X_m_*, *X_m_*, and *X_r_*, respectively. Then, the crossover operation is employed to obtain an intermediate solution *X_c_*, as shown in Equation (16).

(16) $$c_{j}=\left\{\begin{array}{ll} q_{j} & if(p\geq 0.5)\\ g_{j} & if(p<0.5) \end{array}\right.$$

where *c_j_* represents the *j*th dimension of the intermediate solution *X_c_* (*c*_1_, *c*_2_…, *c_D_*). Finally, the fitness of two different solutions *X_c_* and X_m_* is calculated and compared. The subgroup global optimum will be replaced with the new individual if the current best fitness is inferior to the new fitness. The pseudocode below describes the details of the process of BES (shown in Algorithm 3). This step is only used to help optimize the results in the early stage, and the threshold is set to 0.2.
**Algorithm 3.** BES **Input:** the best solution for subpopulation *X*_m_^*^, *MaxIter*, *t*
1 if *t*/*MaxIter* < 0.22 **for** each solution in subpopulation3   Randomly select individual *R* in the population4   Use Equation (15) to calculate the new solution *X*_q_5   Perform crossover between *X*_q_ and *X*_m_^*^ by Equation (16) to get *X*_c_6   if the fitness of *X*_c_ is a better solution, update *X*_m_^*^
7 **end**8 end  **Output:** X_m_^*^

## 5. Framework of MSWOA

**Figure 3:**
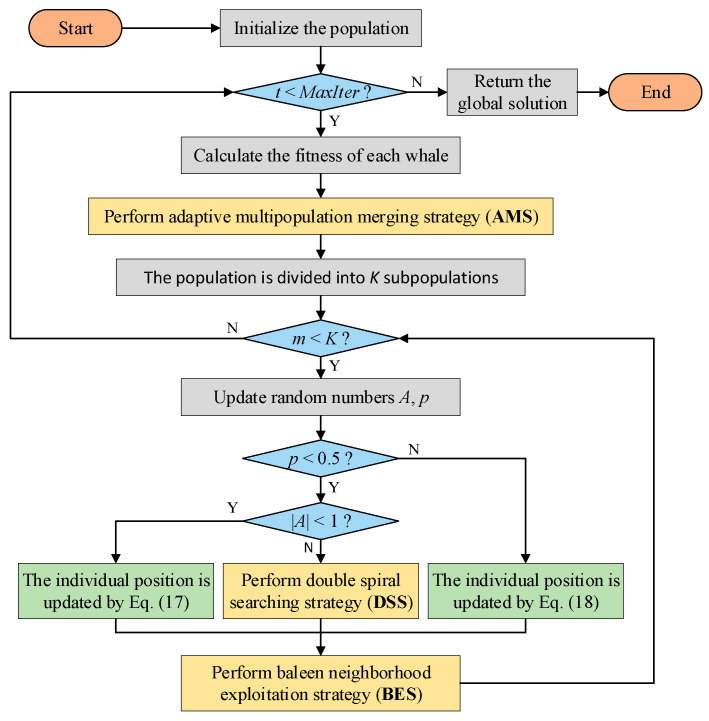
Flowchart of MSWOA.

The pseudocode and flow chart of MSWOA are shown in Algorithm 4 and Figure 3, respectively. The optimization process mainly includes three stages: dividing the population, updating the positions and enhancing the optimal value of the subpopulation. The MSWOA starts with an initial population and then divides it into *K* subpopulations using the AMS approach. Each subswarm has an optimal individual X_m_*. Accordingly, Equations (2) and (6) are revised to Equations (17) and (18).
**Algorithm 4.** MSWOA1 Initializing the population *X_i_* (*i* = 1, 2…, *n*)2 **while** (*t* < *MaxIter*)3  Divide population using AMS 4  **for** each subpopulation5    **for** each solution of subpopulation6       Update *a*, *l*, *p*, *A*, and *C*7       If (*p* < 0.5)8        If (|*A*| < 1)9          Calculate the new position of the current individual according to Equation (17)10         else if (|*A*| > 1)11          Randomly select a solution (*X_rand_*) 12          Calculate the new position of the current individual according to Equation (13)13        end14      else if (*p* > 0.5)15        Calculate the new position of the current individual according to Equation (18)16      end17    **end**18    Update *X*_m_^*^ by BES 19 **end**20 Obtain the global optimal solution X* by comparing *X*_m_^*^ (*m* = 1, 2…, *K*) 21 *t* = *t* + 122 **end**23 return X^*^

(17) $$X_{m}(t+1)=X_{m}^{*}(t)-A\cdot D$$

(18) $$X_{m}(t+1)=D^{\prime }\cdot e^{bl}\cdot \cos(2\pi l)+X_{m}^{*}(t)$$

In the second step, according to the values of the random parameters *p* and *A*, individuals are updated using one formula from Equations (13), (17), or (18). After updating each subpopulation, the BES strategy is conducted to purposefully explore the optimal value. If the fitness value of the obtained solution is better, X_m_* will be updated. The optimal values of *K* subpopulations are compared at the end of each iteration, and the global optimal solution is the search agent with the best fitness value.

The computational complexity of MSWOA depends on the maximum number of iterations (*MaxIter*), subpopulation size (*M*), subpopulation number (*K*), dimension size (*d*), and population size (*N*). The time complexity of the improved algorithm mainly relies on four parts: initialization, Adaptive Multipopulation merging Strategy (AMS), Double Spiral searching Strategy (DSS), and Baleen neighborhood Exploitation Strategy (BES). The initial stage is *O*(*N × d*). The complexity under AMS is *O*(*N*). The adaptability of the DSS is assessed to be *O*(*K × M × d*). The solution update mechanism of BES is *O*(*K × M × d + K × M*). The total time complexity is *O*(*MSWOA*) = *O*(*N × d*) *+O*(*N + K × M × d + K × M*).

### 5.1. Fitness Function

The evaluation of feature subsets is a crucial aspect of wrapper-based approaches. Generally, the evaluation metrics are classification accuracy and the number of features. In this work, the classification accuracy is calculated by the k-nearest Neighbor (*k*-NN) classifier (where *k* = 5) [70]. Since the classification accuracy needs to be maximized while the number of features needs to be minimized, the classification error rate is chosen as an evaluation criterion. In this paper, the fitness function combines the classification error rate and the number of selected features, as shown in Equation (19). Therefore, the criterion is to minimize the fitness value [71].

(19) $$Fitness=\alpha \cdot ErrorRate+\beta \cdot \frac{\left|R\right|}{\left|N\right|}$$

where *ErrorRate* is the classification error rate, *R* is the selected features, and *N* is the full set of features. *α* and *β* are the parameters that regulate the ratio of that classification rate and length of a subset, and β=(1−α) and α∈[0, 1]. In this work, we set *α* = 0.99 [72].

### 5.2. Experimental Results and Discussion

Each experiment is completed using MATLAB2017. The machine used to conduct the experiments is configured with an Intel Core i5-10400 2.90 GHz CPU and 16.0 GB RAM. The evaluator is the *k*-NN classifier, and each dataset is randomly partitioned into a training set and a test set according to the ratio of 80% and 20% [73].

Cross-validation is a more comprehensive model evaluation method that makes effective use of the dataset to enhance the stability and reliability of the model [74]. In comparison to a single train–test split, 5-fold cross-validation divides the entire dataset into five equal parts. In each iteration, one part is used as the validation set, while the remaining four parts are used for training. This process is repeated five times, with a different part serving as the validation set in each iteration. The final model performance is determined by aggregating the results from these five validation rounds. This method helps mitigate the potential evaluation bias introduced by a single validation set and provides a more stable and thorough assessment of model performance [75].

Moreover, cross-validation improves the generalization ability of the model, ensuring that it performs well across different data subsets, thereby reducing the risk of overfitting [76,77,78]. Additionally, it maximizes the utility of each data sample, making it particularly beneficial in cases where the dataset is limited in size. Finally, cross-validation reduces the variability in evaluation results due to the randomness of data splits by repeatedly validating the model on different data partitions, leading to more reliable performance metrics.

For all trials, 5-fold cross-validation is utilized to verify the performance of the selected feature subset to reduce the overfitting issue.

### 5.3. Datasets and Evaluation Metrics

Twenty different benchmark datasets are gathered from the University of California at Irvine (UCI) [79] to assess the performance of the proposed MSWOA. The details of all the datasets are presented in Table 1. The dimensions of these datasets range from 60 to 22,283, and the datasets were collected from various fields, such as biological/gene expression (Lung_Cancer, Leukemia, Prostate_Tumor), physic (Sonar), speech recognition (Isolet), and face images (Yale, orlraws10P, warpPIE10P). Hence, the performance of the MSWOA can be thoroughly investigated.

The experiments use the following evaluation metrics: average fitness values, average classification accuracy, the number of selected features, convergence curves, and run time. The primary performance indicator for assessing the algorithms is the classification accuracy. In addition, we conduct a significance test and use the symbols ‘+’ ‘−’ ‘≈’ to show various differences in the table, where ‘+’ indicates the MSWOA outperforms the competitor algorithm significantly, ‘−’ indicates the competing algorithm greatly outperforms the MSWOA, and ‘≈’ indicates there is no significant difference. The population size is set according to the corresponding papers [72,80,81,82]. The maximum number of iterations is 100 and the experiments are executed 30 times on each dataset. The feature selection threshold is set to 0.5, which means that if the value is more than 0.5, the feature is chosen; otherwise, the feature is not chosen.

### 5.4. Comparison Between the WOA-Based Approaches

To verify the superiority of the proposed MSWOA, it is compared with the original WOA and five recent WOA-based methods, including the RDWOA [83], SBWOA [84], QWOA [85], WOASA [86], and WOACM [87]. Table 2 displays several main parameters of the algorithms that have been well tuned.

The average classification accuracy and standard deviations of seven algorithms for the test sets are reported in Table 3, with the numbers in bold indicating the best results. As seen from Table 3, MSWOA outperforms the other variants on 17 out of the 20 datasets. On the PDC, Nci9, and GLIOMA datasets, WOASA exceeds the other approaches and MSWOA achieves the second-highest classification accuracy. On the SRBCT and Leukemia datasets, QWOA, WOACM, and MSWOA all attain 100% classification accuracy. For the Lung_discrete dataset, MSWOA achieves an accuracy of 94.29%, outperforming the second-best result of 92.38%. Furthermore, significance testing indicates that MSWOA holds significant advantages over competing algorithms on most datasets, including Yale, Lung, and Lung_Cancer. In addition, the significance test results indicate that the MSWOA has apparent advantages over the competitive algorithms on most datasets, such as Yale, Lung, and Lung_Cancer. The excellent performance of MSWOA is mainly due to the proposed AMS technique, which boosts the global search capability to explore the high-performance space. Simultaneously, the BES strategy enhances individual quality in the first 20 iterations, providing a better exploration direction for the population.

The average number of selected features of seven methods on 20 datasets is provided in Table 4. The experiment indicates that MSWOA selects fewer features than other algorithms in 15 of the 20 datasets. RDWOA performs second only to MSWOA, with the best results on 3 datasets (i.e., PDC, warpPIE10P, and Arcene). SBWOA performs best on 2 datasets (i.e., Semion and Isolet). Moreover, the average number of selected features of MSWOA is 364.3767 and it ranks first, followed by RDWOA, SBWOA, WOASA, WOA, WOACM, and QWOA. The AMS strategy is the primary rationale for selecting the smallest number of features. Using the proposed AMS, we can evaluate numerous candidate feature subsets rather than just one in each loop, increasing the probability of finding the best answer. To some extent, the AMS also helps to enhance the classification accuracy. For example, on the GLI_85 dataset, MSWOA selects the fewest features compared to the other algorithms and ranks first with a classification accuracy of 100%. In conclusion, the suggested MSWOA determines the best feature combination with high classification accuracy and very few features.

The fitness value can be used to deeply investigate the robustness of the MSWOA. Table 5 shows the standard deviations and average fitness of the seven methods, from which we can see that the MSWOA achieves a better performance than other six methods on 17 out of the 20 datasets. WOASA obtains the smallest fitness values in the remaining three datasets, and MSWOA is slightly worse. WOACM comes in second out of the 6 datasets and is quite close to MSWOA. In addition, the significance test results demonstrate that MSWOA performs better than other methods on most datasets, particularly when compared with WOA, QWOA, and WOACM. MSWOA performs best on the Semion, Lung, 9_Tumors, and Lung_Cancer datasets, with the other algorithms trailing far behind. From the overall results, MSWOA has outstanding performance on different dimensional datasets, which verifies the excellent robustness of MSWOA.

**Figure 4:**
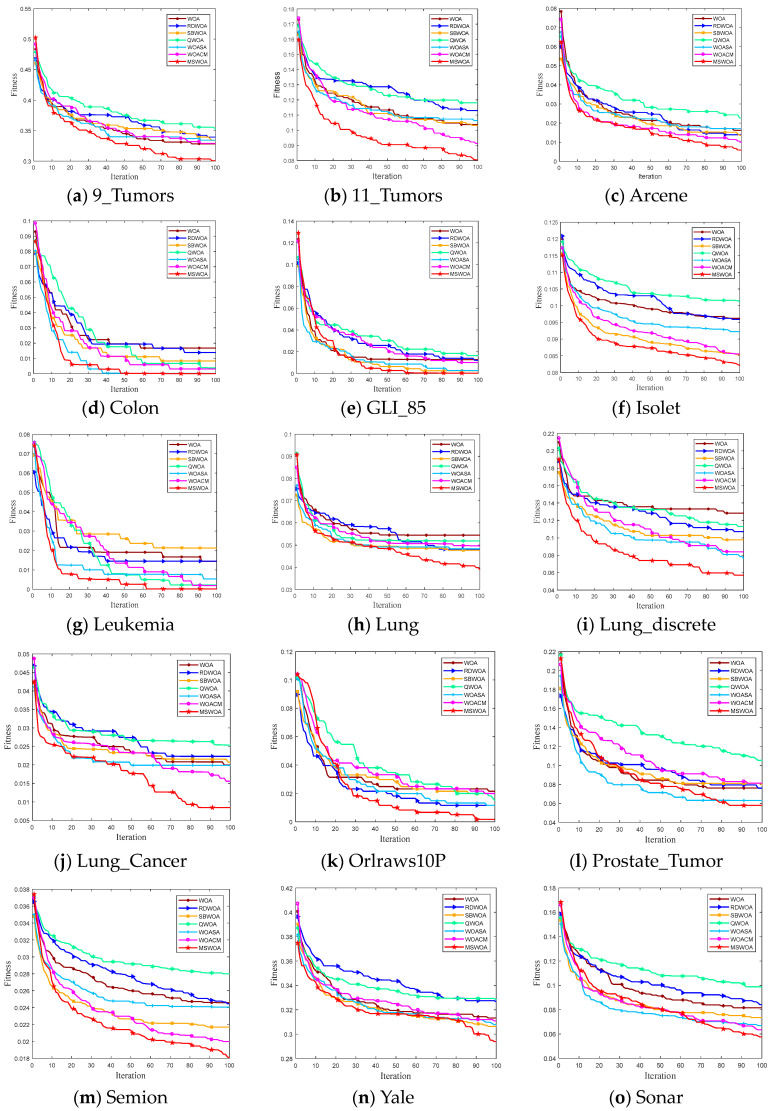

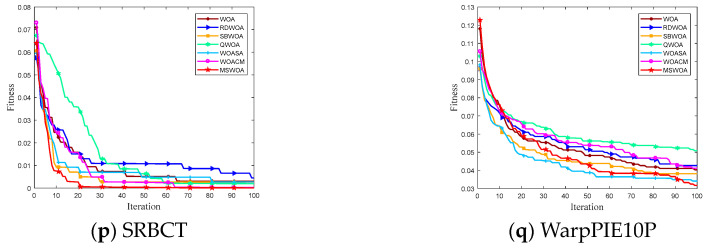
Seven methods’ convergence curves on 17 datasets.

To demonstrate the convergence capability of the proposed MSWOA, the variation curves of the fitness of the seven algorithms on 17 datasets are depicted in Figure 4, from which we can see that the fitness of MSWOA converges fast and achieves the best results in most cases. To explain the change in the mean fitness values, several datasets are presented as examples. For instance, on the Lung_discrete, the fitness value of MSWOA drops to approximately 0.09 after around 25 iterations, whereas the best fitness value among the other six algorithms remains at approximately 0.12. On the 11_Tumors, Isolet, and Lung_discrete datasets, MSWOA converges much faster than the other methods in the entire procedure. On the 9_Tumors, Orlraws10P, and Semion datasets, some algorithms (e.g., WOA, RDWOA, QWOA, SBWOA) perform slightly better than MSWOA in the early phase, but MSWOA can seek better positions and quickly exceeds the others by using the BES strategy. On the Colon, Lung_Cancer, and Yale datasets, MSWOA continuously eliminates search stalls, where the other algorithms are no longer updated. The Leukemia dataset shows that although the other algorithms can also handle the stagnant dilemma, their final fitness values are worse than MSWOA. This is due to the AMS strategy’s enhanced exploration ability, which discoveries additional potential locations.

Training time is a crucial metric for assessing the efficiency of FS algorithms. The average training time for all competing approaches is shown in Table 6. According to the experimental results, the execution time of MSWOA increases slightly when compared with WOA except for the Isolet dataset, because the BES strategy requires one additional estimation of the fitness value. The WOA and RDWOA attain the lowest average training time on nine datasets, respectively. The SBWOA consumes less time on two datasets. According to the average training time, WOA has the fastest calculation speed, followed by SBWOA, RDWOA, MSWOA, WOACM, QWOA, and WOASA. Although RDWOA has the shortest training time on nine datasets, it performs poorly on high-dimensional datasets. In contrast, MSWOA is competitive with the other algorithms on high-dimensional datasets. For example, on the orlraws10P dataset, MSWOA is less than RDWOA, QWOA, WOASA, and WOACM and comparable to SBWOA. Furthermore, it is far superior to the comparison methods in the accuracy, fitness, and screening feature. As a result, MSWOA obtains a more comprehensive feature subset at a reduced cost, especially for high-dimensional datasets.

### 5.5. Comparisons with Other Well-Known Optimization Algorithms

In this subsection, MSWOA is compared with six different metaheuristic methods to further validate its effectiveness, including MPSO [88], HGWOP [89], HGSO [81], DSSA [90], BGOA [80], and HBBOG [91]. Table 7 displays several main parameters of the methods that have been well tuned.

The average classification accuracy rates, standard deviations, and rankings of MPSO, HGWOP, HGSO, DSSA, BGOA, HBBOG, and MSWOA are presented in Table 8, where the numbers in bold indicate the optimal results. As shown in Table 8, MSWOA has the highest classification accuracy on 15 out of the 20 datasets. On the Sonar, Semion, and Isolet datasets, HGWOP takes first place and the significance test results show little difference between HGWOP and MSWOA. HGSO slightly outperforms MSWOA on the Lung_Cancer datasets. BGOA rounds out the top on the Lung dataset, while the proposed MSWOA ranks second. On the SRBCT and GLI_85 datasets, HGSO and BGOA reach the same level of accuracy as MSWOA, respectively. On the Arcene dataset, MSWOA, HGSO, BGOA, and HBBOG achieve close classification accuracies. Furthermore, the results of the significance tests reveal that MSWOA performs better than other comparison methods on most datasets. For example, when compared to DSSA, MSWOA remained significantly different on 17 datasets and performed similarly on 3 datasets.

According to Table 9, MSWOA produces a smaller number of features than other approaches in 15 out of the 20 datasets. HGSO performs slightly better than MSWOA on the warpPIE10P, 9_Tumors, and Prostate_Tumor datasets. On the PDC and GLIOMA datasets, BGOA is superior to MSWOA. The average number of features selected by MSWOA is 362.2713, which is less than that of any other algorithm. Observing the final ranking, the MSWOA achieves the best results, followed by HGSO, BGOA, MPSO, HBBOG, HGWOP, and DSSA. For high-dimensional datasets, the effect of MSWOA is more pronounced. For example, on the Arcene dataset, the average number of features selected by MSWOA is 600.7333, barely 6% of the total features. As a whole, MSWOA can achieve a higher classification accuracy while using fewer features on high-dimensional and complex datasets. The result also demonstrates that the MSWOA has a significant advantage over the other algorithms in terms of the convergence speed. Considering all the results above, MSWOA is a very competitive feature-selection algorithm. Although MSWOA demonstrates advantages in various aspects, such as achieving higher classification accuracy on 15 out of 20 datasets and significantly reducing the number of selected features, its limitations warrant deeper exploration.

Firstly, while the algorithm’s performance generally surpasses other methods, it did not show substantial superiority over HGSO and BGOA on specific datasets like Lung, SRBCT, and GLI_85. This suggests that MSWOA may have room for further optimization when handling high-dimensional data and complex feature distributions.

### 5.6. MSWOA for Human Fall Recognition

#### 5.6.1. Problem Description

In this section, the proposed MSWOA is employed as an FS method in a human fall-detection system to enhance the classification recognition. The fall-detection system allows the elderly to obtain the necessary assistance in time after an accidental fall, reducing injuries caused by falls. Human activities are complex composites composed of various simple motions, influenced by diverse contexts, environments, and individual differences, which contribute to variations in behavior [92,93,94]. Consequently, machine learning models trained on incomplete samples and limited features typically exhibit poor generalization performance, making it difficult to accurately differentiate between fall incidents and routine daily activities. To ensure an adequate dataset and diverse feature types, this chapter outlines the design of a data-collection scheme for fall detection.

The current literature divides fall-detection systems into three broad classes, i.e., wearable device, computer vision, and environmental sensor systems [95]. Nevertheless, the latter two systems have the disadvantage of a limited monitoring range, which results in fall detection failing when the user is not within the sensor coverage region. In addition, a vision-based fall-detection system will bring users a huge risk of privacy disclosure. Therefore, the wearable system is selected for a fall-detection experiment because of the benefits of a low cost, simplicity, and low privacy disclosure risk.

**Figure 5:**
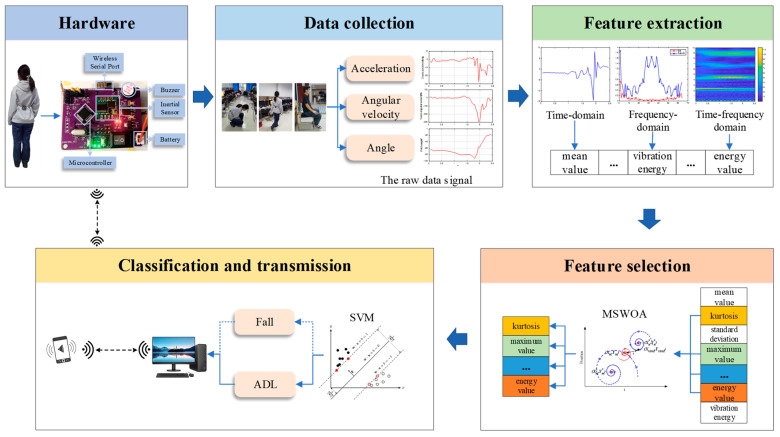
Flowchart of the fall detection system.

Figure 5 illustrates the overall flow of the human fall-detection scheme, including the hardware circuitry, data collection, feature extraction and selection, classification, and results transmission. The hardware consists of a microprocessor, a battery, an inertial sensor module (i.e., MPU6050), a wireless serial module, and an alarm module (i.e., buzzer). MPU6050 has an accelerometer and a gyroscope, which can capture acceleration and angular velocity values and automatically calculate three angles (i.e., pitch, yaw and roll). The sensor’s accelerometer has a measurement range of ±16 g, while the gyroscope’s range is ±2000°/s. The angular range for the X and Z axes is ±180°, and for the Y axis, it is ±90°. The measurement accuracy of the posture angles is 0.05° when static and can reach 0.1° in dynamic conditions, ensuring high measurement precision. To form the original dataset, 13 time-domain features, 6 frequency-domain features, and 8 time-frequency features are extracted from the raw data. Furthermore, the feature subset containing the most information is selected as the input data for the classifier (SVM) using a feature selection method. If the recognition result is a fall, an alert signal is transmitted to both the guardian’s phone and the wearer’s device.

#### 5.6.2. Results and Discussion

**Figure 6:**
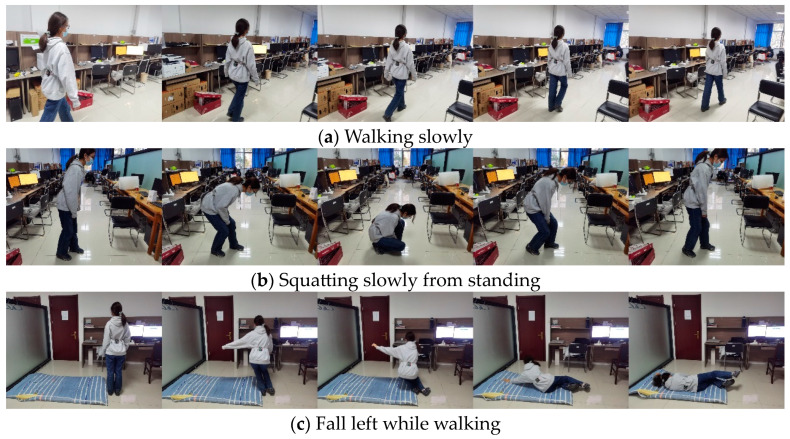
The data collection details of the SFall dataset.

**Figure 7:**
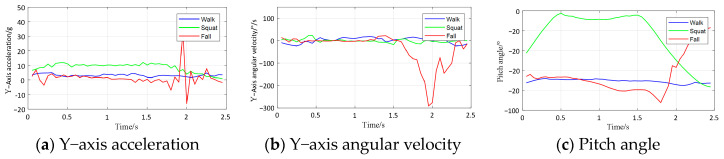
Signal changes in acceleration, angular velocity, and angle for three types of activities.

The dataset used in the experiment is called SFall, which consists of activities of daily living (ADLs) and falls performed by volunteers. The experiment included seven participants—two females and five males. Due to the inherent risk of fall activities, elderly volunteers were not involved, and all participants were students with an average age of 22 years, an average height of 174.7 cm, and an average weight of 65.2 kg. The fall detection system was configured with a sampling frequency of 20 Hz, and each sampling session was set to a duration of 10 s. The SFall dataset comprises 157 collected samples, each with 200 data points and 243 features derived from acceleration, angular velocity, and angular displacement data. Table 10 shows six falls and eight ADLs. These features include 13 time-domain, 6 frequency-domain, and 8 time-frequency domain metrics, extracted across three axes (x, y, z) from MPU6050 sensor data. The volunteers fix the equipment at the waist and perform a set of activities to collect data. Figure 6 depicts the experimental scenario of some of the actions. The sampling frequency for the data-collection experiment is set to 20 Hz. The acquisition time is 10 s for all falls, and recalibration was performed after every session. As shown in Figure 7, the data signals of the Y-axis acceleration, Y-axis angular velocity, and pitch angle for the three behaviors (walk, squat, and fall) have different magnitudes of variation, which indicates that the collected data can extract effective features to separate falls from ADLs.

On the SFall dataset, MSWOA is experimented against three advanced methods to evaluate their performance, including SASS [96], COLSHADE [97], and sCMAgES [98]. To obtain stable results, the test is run 30 times, and the classifier is Support Vector Machine (SVM). The evaluation metrics are the F1-score (FS) and confusion matrix. As shown in Table 11, TP is the number of falls correctly predicted as falls, FP is the number of ADLs incorrectly predicted as falls, FN is the number of falls incorrectly predicted as ADLs, and TN is the number of ADLs correctly predicted as ADLs. According to the experimental results in Table 12, MSWOA has obtained the best performance. Based on the F1-score analysis, MSWOA achieved a score of 89.6567%, which is 1.5 percentage points higher than the second-ranked SASS algorithm. According to the table, the accuracy of MSWOA is the highest, which is 20% higher than sCMAgE. Based on the confusion matrix, MSWOA predicts the highest number of correct falls with 12.2 and the least number of overall false predictions (FN+FP) with 2.8, indicating that the algorithm possesses robust recognition capabilities for both fall and non-fall behaviors. Thus, the MSWOA-enabled human fall detection system has more robust recognition performance, which proves that the MSWOA method has promising applications in realistic optimization problems.

## 6. Conclusions

Because of the insufficient randomness of the exploration formula and the single leader, WOA-based feature-selection techniques often suffer from premature convergence and entrapment in local optimal solutions. Therefore, a multispiral WOA (MSWOA) for FS is proposed in this paper to address these dilemmas. The MSWOA includes three major improved modifications. Initially, we utilized an Adaptive Multipopulation merging Strategy (AMS) with a novel allocation method for population delimitation. Each subpopulation chooses its best individual to be the leader, which expands the exploration space and diversity. Additionally, using the Double Spiral updating Strategy (DSS), individuals take their current position as a starting point and randomly explore outward along the spiral trajectory. In this case, the search process is not limited by the distance between the individuals, which prevents search stagnation in the later iterations. Finally, a Baleen neighborhood Exploitation Strategy (BES) with quadratic interpolation was incorporated into the early search phase to obtain better positions and facilitate convergence by searching for regions near the optimal search agent. The proposed MSWOA approach was evaluated against the original WOA and 11 other recent algorithms, including 5 WOA-based and 6 metaheuristic variants on 20 UCI datasets. The results demonstrated that the MSWOA has advantages in classification accuracy, the number of selected features, and the fitness values on most datasets. In addition, MSWOA exhibited a faster convergence speed when compared to the competitive methods. Moreover, the MSWOA is employed for feature selection in a human fall-detection task, and experimental results indicated that it is capable of solving real-world problems.

There is further work that can be performed in the future, for example, combining the multipopulation technique with other updating mechanisms or utilizing different classifiers (e.g., neural networks) to further evaluate the algorithm’s effectiveness. Moreover, the temporal complexity of the algorithm needs to be further reduced to achieve greater computational efficiency. The new MSWOA algorithm can also be applied to other engineering fields, such as fault diagnosis, image segmentation, and path planning.

## Figures and Tables

**Table 1:** List of datasets used in the experiments.

No.	Dataset	Classes	Number of Features	Number of Instances
1	Sonar	2	60	208
2	Semion	10	265	1593
3	Lung_discrete	7	325	73
4	Isolet	26	617	1560
5	PDC	2	756	753
6	Yale	15	1024	165
7	Colon	2	2000	62
8	SRBCT	4	2308	83
9	warpPIE10P	10	2420	210
10	Lung	5	3312	203
11	GLIOMA	4	4434	50
12	9_Tumors	9	5726	60
13	Leukemia	2	7070	72
14	Nci9	9	9712	60
15	Arcene	2	10,000	200
16	orlraws10P	10	10,304	100
17	Prostate_Tumor	2	10,509	102
18	11_Tumors	11	12,533	174
19	Lung_Cancer	5	12,600	203
20	GLI_85	2	22,283	85

**Table 2:** Each optimization algorithm’s parameter settings.

Algorithm	Parameter Settings
WOA	The value of a ranges from 2 to 0, b is 1
RDWOA	The value of a ranges from 2 to 0, b is 1, w1 and w2 are the random numbers in (0, 1) and (0.5, 1)
SBWOA	The value of a1 ranges from 2 to 0, b is 1
QWOA	q = 2 − t × 2/maxGen, *p* = 1
WOASA	The value of a ranges from 2 to 0, b is 1, T0 = 2 ×|N|
WOACM	The value of a ranges from 2 to 0, b is 1, the value of r ranges from 0.9 to 0
MSWOA	The value of a ranges from 2 to 0, b is 1, γ = 0.8

**Table 3:** The seven algorithms’ average classification accuracy on the 20 datasets.

Dataset	Criteria	WOA	RDWOA	SBWOA	QWOA	WOASA	WOACM	MSWOA
Sonar	Mean	92.1138 (+)	91.7886 (+)	92.8485 (+)	90.4878 (+)	93.5772 (≈)	93.9024 (≈)	**94.3902**
	Std	2.0584	2.9189	2.9480	1.3135	2.6332	2.1587	1.9049
Semion	Mean	97.9769 (+)	97.9036 (+)	98.1447 (+)	97.6520 (+)	97.9874 (+)	98.3962 (≈)	**98.4801**
	Std	0.3693	0.4894	0.4007	0.2399	0.3595	0.2853	0.4226
Lung_discrete	Mean	87.1429 (+)	89.2857 (+)	90.2381 (+)	88.8095 (+)	92.3810 (≈)	91.6667 (+)	**94.2857**
	Std	3.8686	4.0195	5.0451	3.5395	4.8562	4.1582	4.2857
Isolet	Mean	90.8440 (+)	90.7799 (+)	91.7628 (≈)	90.4380 (+)	91.2607 (+)	91.8483 (≈)	**92.1368**
	Std	0.8386	1.1124	0.7814	0.6253	0.6407	0.7387	0.8097
PDC	Mean	83.6424 (+)	84.4592 (≈)	83.9294 (+)	82.3620 (+)	**85.9603** (≈)	83.5320 (+)	85.2318
	Std	2.1639	2.0713	2.5127	2.3099	2.0116	3.5488	2.0953
Yale	Mean	68.7879 (+)	67.2727 (+)	69.2929 (≈)	67.4747 (+)	69.2929 (+)	68.9899 (+)	**70.5051**
	Std	2.9432	3.3562	2.5633	1.3401	2.3122	2.4347	2.7028
Colon	Mean	98.3333 (+)	98.6111 (≈)	99.1667 (≈)	99.7222 (≈)	**100** (≈)	99.7222 (≈)	**100**
	Std	3.3333	3.1056	2.5000	1.4959	0	1.4959	0
SRBCT	Mean	99.7917 (≈)	99.5833 (≈)	99.7917 (≈)	**100** (≈)	99.7917 (≈)	**100** (≈)	**100**
	Std	1.1219	1.5590	1.1219	0	1.1219	0	0
warpPIE10P	Mean	96.2698 (≈)	95.8730 (+)	85.4762 (+)	95.3968 (+)	96.8254 (≈)	96.2698 (≈)	**96.9841**
	Std	1.8115	1.9375	5.0076	1.0529	1.5471	1.4656	1.2192
Lung	Mean	94.5833 (+)	95.1667 (+)	95.2482 (+)	95.0000 (+)	95.1667 (+)	95.0833 (+)	**96.0833**
	Std	1.4554	1.1055	1.2615	0	0	0.4488	1.3969
GLIOMA	Mean	91.0000 (+)	92.6667 (≈)	91.6561 (+)	88.3333 (+)	**95.0000** (≈)	90.3333 (+)	94.3333
	Std	5.9722	5.1208	5.6491	4.5338	5.0000	6.0461	4.9554
9_Tumors	Mean	66.9444 (≈)	65.8333 (+)	45.5556 (+)	64.7222 (+)	66.3889 (+)	66.9444 (≈)	**69.4444**
	Std	5.8860	5.4220	9.0608	3.5246	4.5559	5.0384	4.4790
Leukemia	Mean	98.5714 (+)	98.5714 (+)	97.8571 (+)	**100** (≈)	99.5238 (≈)	**100** (≈)	**100**
	Std	2.8571	2.8571	3.2733	0	1.7817	0	0
Nci9	Mean	60.5556 (≈)	60.2778 (≈)	60.2778 (≈)	58.8889 (+)	**61.6667** (≈)	59.7222 (≈)	60.8333
	Std	4.7791	5.1295	4.1295	2.0787	4.6148	3.1056	3.8188
Arcene	Mean	89.7500 (+)	90.0833 (≈)	90.0833 (≈)	87.9167 (+)	90.2500 (≈)	89.6667 (≈)	**90.9167**
	Std	2.2684	2.4566	2.4566	1.4554	2.6887	2.7183	1.8801
orlraws10P	Mean	97.8333 (+)	98.8333 (≈)	98.0000 (+)	98.5000 (+)	98.8333 (≈)	98.0000 (+)	**99.6667**
	Std	2.7938	2.1148	2.7689	2.2913	2.1148	2.4495	1.2472
Prostate_Tumor	Mean	92.3333 (+)	92.3333 (≈)	92.0000 (+)	89.5000 (+)	93.6667 (≈)	91.8333 (+)	**94.1667**
	Std	3.5901	3.3500	3.5590	2.9861	3.6362	3.0231	3.4359
11_Tumors	Mean	89.9020 (+)	88.8235 (+)	89.8039 (+)	88.5294 (+)	89.6078 (+)	91.0784 (≈)	**92.0588**
	Std	2.4822	2.8818	2.2442	1.1642	2.4880	2.2119	2.1680
Lung_Cancer	Mean	98.0833 (+)	97.8333 (+)	98.0000 (+)	97.6667 (+)	98.0833 (+)	98.5000 (+)	**99.1667**
	Std	1.0574	1.0672	1.0000	0.6236	1.2388	1.2247	1.1785
GLI_85	Mean	98.8235 (+)	98.8235 (+)	85.0980 (+)	98.6275 (+)	99.8039 (≈)	99.2157 (≈)	**100**
	Std	2.3529	2.3529	8.4200	2.4880	1.0559	1.9996	0
1st/2nd		0/2	0/3	0/1	2/1	4/**8**	2/7	**17**/3
+/−/≈		16/0/4	12/0/8	14/0/6	17/0/3	7/0/13	8/0/12	

**Table 4:** The average number of selected features of seven methods on 20 datasets.

Dataset	Full	WOA	RDWOA	SBWOA	QWOA	WOASA	WOACM	MSWOA
Sonar	60	21.2667	16.9000	16.2333	27.4000	20.5333	18.2667	**12.2333**
Semion	265	120.9667	98.3000	**88.1667**	126.1333	109.3333	109.1333	88.3000
Lung_discrete	325	32.9667	26.6000	39.3333	68.4000	28.1000	51.4667	**20.8667**
Isolet	617	346.1333	283.9333	**222.3000**	396.4000	351.1000	295.5000	263.2667
PDC	756	62.3000	**34.0333**	47.9000	165.9667	39.4667	152.9667	77.7000
Yale	1024	436.2333	253.2667	241.6000	633.1000	381.4333	402.4333	**201.9667**
Colon	2000	50.5000	15.1000	30.0000	231.2667	42.0000	62.2667	**14.9667**
SRBCT	2308	229.7000	90.8333	138.4000	449.4667	157.6667	55.8000	**26.8667**
warpPIE10P	2420	818.9333	**447.6667**	500.4333	1260.2333	652.3667	1041.6000	457.7000
Lung	3312	264.5000	112.0333	217.6667	790.0667	165.4667	349.1000	**77.3000**
GLIOMA	4434	66.0333	39.5667	95.3667	313.5333	54.8000	291.1333	**36.4667**
9_Tumors	5726	709.2333	785.8667	802.9333	2078.8333	931.0000	1160.5333	**668.6333**
Leukemia	7070	161.6667	251.8000	102.3667	1525.5333	426.9667	1357.2000	**96.0667**
Nci9	9712	879.8667	359.4667	880.7667	2972.5000	495.9333	1616.2667	**324.4667**
Arcene	10,000	1378.0333	**787.9667**	985.4333	4292.7000	1402.1333	2573.1667	1347.0333
orlraws10P	10,304	38.0333	36.1000	42.7333	129.6667	42.2667	192.8333	**24.6667**
Prostate_Tumor	10,509	244.4333	194.2333	195.7333	1467.5333	396.1667	476.9000	**80.1000**
11_Tumors	12,533	4783.4333	2928.9000	2834.1000	5626.1333	4392.5667	3760.3667	**2437.4000**
Lung_Cancer	12,600	1267.4333	1067.0333	1142.4333	2808.7000	1096.9333	888.5667	**229.1000**
GLI_85	22,283	2513.8333	951.0333	1337.0667	6408.5667	1926.4667	5108.0000	**802.4333**
Ave	6101	721.2750	439.0317	498.0483	1588.6067	655.6350	998.1750	**364.3767**
Rank		5	2	3	7	4	6	1

**Table 5:** The seven algorithms’ average fitness values on 20 datasets.

Dataset	Criteria	WOA	RDWOA	SBWOA	QWOA	WOASA	WOACM	MSWOA
Sonar	Mean	0.0816 (+)	0.0841 (+)	0.0735 (+)	0.0987 (+)	0.0670 (≈)	0.0634 (≈)	**0.0576**
	Std	0.0208	0.0292	0.0254	0.0128	0.0267	0.0216	0.0188
Semion	Mean	0.0246 (+)	0.0245 (+)	0.0217 (+)	0.0280 (+)	0.0241 (+)	0.0209 (+)	**0.0039**
	Std	0.0041	0.0049	0.0041	0.0024	0.0200	0.0181	0.0246
Lung_discrete	Mean	0.1283 (+)	0.1069 (+)	0.0979 (+)	0.1129 (+)	0.0763 (≈)	0.0841 (+)	**0.0572**
	Std	0.0383	0.0397	0.0498	0.0353	0.0481	0.0415	0.0424
Isolet	Mean	0.0963 (+)	0.0959 (+)	0.0852 (≈)	0.1011 (+)	0.0922 (+)	0.0855 (≈)	**0.0821**
	Std	0.0089	0.0114	0.0079	0.0069	0.0073	0.0077	0.0081
PDC	Mean	0.1628 (+)	0.1543 (≈)	0.1597 (+)	0.1768 (+)	**0.1395 (≈)**	0.1651 (+)	0.1457
	Std	0.0223	0.0203	0.0251	0.0248	0.0200	0.0364	0.0206
Yale	Mean	0.3133 (+)	0.3265 (+)	0.3064 (≈)	0.3282 (+)	0.3077 (+)	0.3109 (+)	**0.2940**
	Std	0.0300	0.0334	0.0257	0.0131	0.0235	0.0245	0.0272
Colon	Mean	0.0168 (+)	0.0138 (+)	0.0084 (+)	0.0039 (+)	0.0002 (≈)	0.0031 (≈)	**0.0001**
	Std	0.0329	0.0307	0.0247	0.0147	0.0008	0.0148	0.0002
SRBCT	Mean	0.0031 (+)	0.0045 (+)	0.0027 (+)	0.0019 (+)	0.0027 (+)	0.0002 (+)	**0.0001**
	Std	0.0118	0.0156	0.0111	0.0020	0.0113	0.0002	0.0001
warpPIE10P	Mean	0.0403 (+)	0.0427 (+)	0.0382 (≈)	0.0508 (+)	0.0341 (≈)	0.0412 (+)	0.0317
	Std	0.0192	0.0192	0.0162	0.0102	0.0157	0.0148	0.0127
Lung	Mean	0.0544 (+)	0.0482 (+)	0.0477 (+)	0.0519 (+)	0.0483 (+)	0.0497 (+)	**0.0390**
	Std	0.0143	0.0110	0.0076	0.0021	0.0090	0.0048	0.0139
GLIOMA	Mean	0.0892 (+)	0.0727 (≈)	0.0827 (+)	0.1162 (+)	**0.0496 (≈)**	0.0964 (+)	0.0562
	Std	0.0592	0.0507	0.0631	0.0449	0.0496	0.0599	0.0491
9_Tumors	Mean	0.3285 (+)	0.3396 (+)	0.3342 (+)	0.3529 (+)	0.3344 (+)	0.3293 (+)	**0.3009**
	Std	0.0583	0.0537	0.0501	0.0353	0.0453	0.0499	0.0397
Leukemia	Mean	0.0144 (+)	0.0145 (+)	0.0214 (+)	0.0022 (+)	0.0053 (+)	0.0019 (+)	**0.0001**
	Std	0.0282	0.0281	0.0323	0.0029	0.0175	0.0016	0.0002
Nci9	Mean	0.3914 (≈)	0.3936 (≈)	0.3942 (+)	0.4101 (+)	**0.3800 (≈)**	0.4004 (+)	0.3881
	Std	0.0476	0.0507	0.0412	0.0208	0.0459	0.0309	0.0377
Arcene	Mean	0.1029 (≈)	0.0990 (≈)	0.0992 (≈)	0.1239 (+)	0.0979 (≈)	0.1049 (+)	**0.0913**
	Std	0.0234	0.0245	0.0243	0.0150	0.0274	0.0281	0.0192
orlraws10P	Mean	0.0215 (+)	0.0116 (+)	0.0198 (+)	0.0150 (+)	0.0116 (+)	0.0200 (+)	**0.0017**
	Std	0.0277	0.0209	0.0274	0.0227	0.0210	0.0244	0.0089
Prostate_Tumor	Mean	0.0761 (+)	0.0761 (+)	0.0794 (+)	0.1053 (+)	0.0631 (≈)	0.0813 (+)	**0.0578**
	Std	0.0358	0.0332	0.0353	0.0302	0.0366	0.0303	0.0340
11_Tumors	Mean	0.1038 (+)	0.1130 (+)	0.1032 (+)	0.1180 (+)	0.1064 (+)	0.0913 (+)	**0.0806**
	Std	0.0252	0.0286	0.0223	0.0117	0.0251	0.0218	0.0214
Lung_Cancer	Mean	0.0200 (+)	0.0223 (+)	0.0207 (+)	0.0253 (+)	0.0198 (+)	0.0156 (+)	**0.0084**
	Std	0.0109	0.0108	0.0103	0.0069	0.0126	0.0126	0.0117
GLI_85	Mean	0.0128 (+)	0.0121 (≈)	0.0025 (≈)	0.0165 (+)	0.0028 (≈)	0.0101 (+)	**0.0004**
	Std	0.0230	0.0232	0.0104	0.0246	0.0105	0.0198	0.0004
1st/2nd		0/1	0/1	0/4	0/0	3/5	0/**6**	**17**/3
+/−/≈		18/0/2	15/0/5	15/0/5	20/0/0	10/0/10	17/0/3	

**Table 6:** The average training time of seven algorithms on 20 datasets (unit: s).

Dataset	WOA	RDWOA	SBWOA	QWOA	WOASA	WOACM	MSWOA
Sonar	27.0767	**24.3779**	27.5940	27.4059	56.0842	28.1668	34.1727
Semion	170.6904	**124.2816**	127.9725	218.3407	316.6633	168.6442	167.3333
Lung_discrete	28.9162	**25.0667**	29.5453	29.3761	61.9578	31.5940	38.9689
Isolet	156.7579	112.1603	**105.6040**	169.6362	295.2245	140.4256	153.0561
PDC	60.7638	**44.0457**	57.4741	133.1237	117.2157	107.2577	91.1299
Yale	29.9337	**26.2778**	30.1693	32.5840	63.1351	31.9019	36.0841
Colon	25.1746	**23.5007**	25.9836	27.6154	56.6117	28.9731	33.7010
SRBCT	27.5095	**25.7350**	28.7676	30.2235	58.3703	29.7303	35.1542
warpPIE10P	60.8981	**44.6591**	51.3634	82.9210	114.6850	71.0751	69.6742
Lung	31.1717	**29.0770**	31.9499	46.0428	63.2751	38.8779	39.8671
GLIOMA	**25.6905**	28.5720	28.2346	29.0031	55.6681	29.4977	34.5528
9_Tumors	**41.2546**	46.8909	44.9211	52.0716	83.9295	45.1787	51.3264
Leukemia	**43.1709**	56.4489	49.6950	59.7959	93.7520	58.4227	59.9864
Nci9	**53.1048**	71.8140	64.5118	75.3016	113.1753	69.0995	74.5359
Arcene	**81.5805**	95.9885	93.7707	177.8997	155.1782	125.6167	103.2747
orlraws10P	**26.7257**	48.8303	37.1408	41.0257	58.5471	38.0248	37.9534
Prostate_Tumor	**28.9446**	50.4748	38.3373	51.3003	63.7679	40.2162	41.2868
11_Tumors	78.0815	85.4132	**72.7280**	110.5995	157.1344	80.0640	78.6899
Lung_Cancer	**90.6418**	117.6249	105.0547	234.5773	181.9730	140.6153	110.4799
GLI_85	**63.3169**	160.1452	97.7187	123.5128	122.6435	96.8682	84.6735
Ave	**54.9535**	59.3861	54.8900	83.8468	109.5121	67.0257	65.8194
Rank	1	3	2	6	7	5	4

**Table 7:** Each optimization algorithm’s parameter settings.

Algorithm	Parameter Settings
MPSO	C1 and C2 is 2, r1 and r2 ~ U (0,1), wmin is 0.4, wmax is 0.9
HGWOP	The maximum and minimum values of a are 2 and 0, Crmax is 1, Crmin is 0
HGSO	K = α = β = 1, l1, l2 and l3 is 5 × 10^−3^, 1 × 10^2^ and 1 × 10^−2^, M1 is 0.1, M2 is 0.2,
DSSA	The maximum number of iterations of LSA is 10, c2 and c3 are chosen randomly in [0, 1]
BGOA	The maximum and minimum values of c are 1 and 0.00004, r linearly decreases from 0.9 to 0
HBBOG	a0 = 2, I = 1, E = 1
MSWOA	The value of a ranges from 2 to 0, b is 1, γ = 0.8

**Table 8:** The average accuracy of seven algorithms on 20 datasets.

Dataset	Criteria	MPSO	HGWOP	HGSO	DSSA	BGOA	HBBOG	MSWOA
Sonar	Mean	95.9350 (≈)	**96.7480** (≈)	94.3089 (+)	96.0163 (≈)	94.7967 (+)	96.1789 (≈)	96.6667
	Std	2.0244	2.1201	1.7054	1.6035	1.3701	1.8557	1.4747
Semion	Mean	99.0461 (≈)	**99.2348** (≈)	98.6478 (+)	99.0776 (≈)	98.7107 (+)	99.0566 (≈)	99.1300
	Std	0.2221	0.2654	0.2171	0.3138	0.2483	0.2148	0.3781
Lung_discrete	Mean	93.0952 (+)	92.1429 (+)	93.5714 (+)	92.8571 (+)	92.8571 (+)	92.6190 (+)	**96.6667**
	Std	2.2462	2.1429	2.1429	1.8443	2.6082	2.2462	3.5635
Isolet	Mean	89.6368 (+)	**90.6090** (≈)	88.6004 (+)	89.8825 (+)	89.2735 (+)	89.3697 (+)	90.3526
	Std	0.6791	0.9109	0.4584	0.8095	0.6607	0.8361	0.6842
PDC	Mean	77.3951 (+)	75.6291 (+)	82.2958 (+)	75.7174 (+)	81.5453 (+)	76.5563 (+)	**83.9073**
	Std	3.1144	2.0043	1.3238	1.3500	2.0642	1.9912	2.1436
Yale	Mean	65.4545 (+)	65.8586 (+)	64.9495 (+)	65.4545 (+)	65.7576 (+)	65.5556 (+)	**68.4848**
	Std	2.0101	2.8138	2.4347	2.4242	3.5983	2.1404	3.7196
Colon	Mean	95.2778 (+)	91.1111 (+)	98.0556 (≈)	91.6667 (+)	96.3889 (+)	91.6667 (+)	**99.1667**
	Std	4.1295	2.9918	3.5246	0.0000	4.1295	0.0000	2.5000
SRBCT	Mean	99.7917 (≈)	98.5417 (+)	**100** (≈)	97.7083 (+)	99.3750 (+)	97.2917 (+)	**100**
	Std	1.1219	2.6435	0	3.0118	1.8750	3.0971	0
warpPIE10P	Mean	93.3333 (+)	93.3333 (+)	93.4127 (≈)	93.7302 (+)	93.1746 (+)	93.3333 (+)	**94.127**
	Std	1.4286	0.9524	1.1798	1.1474	1.1878	1.1336	1.3375
Lung	Mean	97.5000 (+)	97.5000 (+)	97.5833 (≈)	97.5000 (+)	**97.7500** (≈)	97.5000 (+)	97.6667
	Std	0	0	0.4488	0	0.7500	0	0.6236
GLIOMA	Mean	83.3333 (+)	80.0000 (+)	87.0000 (+)	80.0000 (+)	86.0000 (+)	80.0000 (+)	**91.3333**
	Std	4.7140	0.0000	5.2599	0.0000	5.5377	0.0000	4.2687
9_Tumors	Mean	66.3889 (+)	65.0000 (+)	65.5556 (+)	63.6111 (+)	65.5556 (+)	65.0000 (+)	**70.0000**
	Std	5.0384	5.8531	4.1574	4.0158	5.1520	3.9675	4.0825
Leukemia	Mean	93.3333 (+)	91.4286 (+)	98.0952 (+)	92.1429 (+)	96.4286 (+)	92.1429 (+)	**100**
	Std	4.0963	4.2857	3.1587	2.1429	4.0195	2.1429	0
Nci9	Mean	61.3889 (+)	57.7778 (+)	66.1111 (+)	58.0556 (+)	66.6667 (+)	57.7778 (+)	**74.7222**
	Std	6.2670	3.6851	4.7791	2.6206	7.4536	3.6851	6.6260
Arcene	Mean	95.4167 (+)	95.6667 (+)	96.1667 (≈)	95.9167 (+)	95.7500 (≈)	95.8333 (≈)	**96.5833**
	Std	1.4554	1.1055	1.4044	1.2047	1.6008	1.3437	1.3668
orlraws10P	Mean	97.5000 (+)	93.5000 (+)	99.6667 (≈)	93.1667 (+)	98.0000 (+)	93.5000 (+)	**100**
	Std	2.8137	2.2913	1.2472	2.4095	2.7689	2.2913	0
Prostate_Tumor	Mean	96.8333 (+)	97.1667 (+)	97.5000 (+)	96.3333 (+)	96.0000 (+)	95.1667 (+)	**98.6667**
	Std	3.2872	2.7938	2.5000	2.5604	3.0000	3.5316	2.2111
11_Tumors	Mean	91.3725 (+)	92.3529 (+)	91.7647 (+)	91.9608 (+)	91.2745 (+)	92.1569 (+)	**93.4314**
	Std	1.3006	1.4409	1.4003	1.9996	1.4173	1.3865	1.4575
Lung_Cancer	Mean	97.0000 (≈)	97.0000 (≈)	**97.4167** (≈)	96.6667 (≈)	97.2500 (≈)	97.2500 (≈)	97.3333
	Std	1.0000	1.0000	0.4488	1.3437	0.9895	0.7500	0.8975
GLI_85	Mean	93.7255 (+)	92.7451 (+)	99.8039 (≈)	92.5490 (+)	**100** (≈)	92.5490 (+)	**100**
	Std	3.9992	2.9149	1.0559	3.3735	0	3.3735	0
1st/2nd		0/2	3/2	2/9	0/1	2/1	0/0	15/5
+/−/≈		16/0/4	16/0/4	12/0/8	17/0/3	16/0/4	16/0/4	

**Table 9:** The average number of selected features of seven methods on 20 datasets.

Dataset	Full	MPSO	HGWOP	HGSO	DSSA	BGOA	HBBOG	MSWOA
Sonar	60	32.4000	26.4667	20.4667	26.5667	41.4000	26.4667	**18.6667**
Semion	265	148.6333	118.6000	115.9333	123.2000	156.2667	124.3667	**96.2667**
Lung_discrete	325	95.3000	132.0000	26.7667	135.8667	109.3667	133.8667	**26.7333**
Isolet	617	394.9667	303.5000	264.6667	304.7000	415.4000	303.9333	**248.6333**
PDC	756	337.1333	363.3000	28.5667	373.8000	**25.8667**	365.6000	80.1667
Yale	1024	522.8000	491.7333	217.8667	503.8000	411.1667	494.7333	**197.1000**
Colon	2000	247.7667	895.6333	20.3000	928.9333	138.4000	879.7333	**17.3667**
SRBCT	2308	500.6667	1068.3333	51.5000	1093.0667	190.8333	1015.6333	**31.7667**
warpPIE10P	2420	1229.7333	1178.2000	**380.8333**	1185.4333	838.9000	1181.3333	426.9000
Lung	3312	458.7667	1500.8667	98.4000	1550.4000	547.6667	1439.7333	**53.7333**
GLIOMA	4434	188.7000	1955.5333	45.9667	2042.8000	**31.2333**	1806.1667	53.0667
9_Tumors	5726	2242.3000	2763.1000	**542.6333**	2804.8333	1186.1667	2721.3667	781.9667
Leukemia	7070	1820.9333	3416.3667	82.2667	3464.1000	173.1000	3392.6000	**27.5000**
Nci9	9712	2244.6667	4716.4333	143.7667	4795.6333	498.7667	4739.6000	**135.5000**
Arcene	10,000	2874.4000	4783.6000	1113.5000	4914.1000	1526.0667	4774.9333	**600.7333**
orlraws10P	10,304	983.8667	4942.8667	79.5667	5037.4333	113.7333	4846.6333	**36.0000**
Prostate_Tumor	10,509	3066.5333	5148.5667	**593.1000**	5209.8333	855.4667	5109.8000	725.6667
11_Tumors	12,533	5077.1667	6126.4000	3234.7000	6209.1333	4939.6333	6056.0000	**2443.2333**
Lung_Cancer	12,600	4629.5000	6030.8667	1170.1333	6182.4333	4933.3000	5834.4667	**1091.5333**
GLI_85	22,283	5374.1333	10,916.9667	205.5667	11,047.0000	444.3333	10,751.2333	**168.7667**
Ave	6101	1620.3404	2847.5277	420.9598	2900.6781	876.9085	2802.1370	**362.2713**
Rank		4	6	2	7	3	5	1

**Table 10:** The falls and ADLs of the SFall dataset.

No.	Cat.	Description	Acquisition Duration (Second)
1	ADL	Walking slowly	10
2	ADL	Jogging	10
3	ADL	Slowly sit down and stand up again	10
4	ADL	Bending slowly from standing and stand straight again	10
5	ADL	Slowly squat and then stand up	10
6	ADL	Lie down slowly and then stand up	10
7	ADL	Walking upstairs and downstairs slowly	10
8	ADL	Mix of daily activities	10
9	Fall	Fall forward while walking	
10	Fall	Fall backward while walking	
11	Fall	Fall left while walking	
12	Fall	Fall right while walking	
13	Fall	Vertical fall while walking	
14	Fall	Fall while jogging	

**Table 11:** Structure of the confusion matrix.

Confusion Matrix		Prediction	
		Falls (P)	ADLs (N)
Reference	Falls (P)	TP	FN
	ADLs (N)	FP	TN

**Table 12:** Recognition results of the four algorithms on the SFall dataset.

Algorithms	FS (%)	TP	FN	FP	FN+FP	Mean Accuracy
MSWOA	**89.6567**	**12.2**	1.6	**1.2**	**2.8**	81.33%
SASS	88.1198	11.1	0.5	2.4	2.9	79.29%
COLSHADE	80.6183	9.4	**0.1**	4.2	4.3	68.61%
sCMAgES	76.4312	11.5	4.9	2.2	7.1	61.83%

## Data Availability

The data are not publicly available due to privacy. The data presented in this study are available on request from the corresponding author.
